# Beamforming Based Full-Duplex for Millimeter-Wave Communication

**DOI:** 10.3390/s16071130

**Published:** 2016-07-21

**Authors:** Xiao Liu, Zhenyu Xiao, Lin Bai, Jinho Choi, Pengfei Xia, Xiang-Gen Xia

**Affiliations:** 1 School of Electronic and Information Engineering, Beihang University, Beijing 100191, China; eeliuxiao@buaa.edu.cn (X.L.); l.bai@buaa.edu.cn (L.B.); 2 Collaborative Innovation Center of Geospatial Technology, Wuhan 430079, China; 3 Beijing Key Laboratory for Network-Based Cooperative Air Traffic Management, and Beijing Laboratory for General Aviation Technology, Beijing 100191, China; 4 School of Electrical Engineerng and Computer Science, Gwangju Institute of Science and Technology (GIST), Gwangju 61005, Korea; jchoi0114@gist.ac.kr; 5 School of Electronics and Information Engineering and the Key Laboratory of Embedded System and Service Computing, Tongji University, Shanghai 200092, China; pengfei.xia@gmail.com; 6 Department of Electrical and Computer Engineering, University of Delaware, Newark, DE 19716, USA; xianggen@udel.edu

**Keywords:** full duplex, self-interference cancellation, beamforming, millimeter-wave, mmWave

## Abstract

In this paper, we study beamforming based full-duplex (FD) systems in millimeter-wave (mmWave) communications. A joint transmission and reception (Tx/Rx) beamforming problem is formulated to maximize the achievable rate by mitigating self-interference (SI). Since the optimal solution is difficult to find due to the non-convexity of the objective function, suboptimal schemes are proposed in this paper. A low-complexity algorithm, which iteratively maximizes signal power while suppressing SI, is proposed and its convergence is proven. Moreover, two closed-form solutions, which do not require iterations, are also derived under minimum-mean-square-error (MMSE), zero-forcing (ZF), and maximum-ratio transmission (MRT) criteria. Performance evaluations show that the proposed iterative scheme converges fast (within only two iterations on average) and approaches an upper-bound performance, while the two closed-form solutions also achieve appealing performances, although there are noticeable differences from the upper bound depending on channel conditions. Interestingly, these three schemes show different robustness against the geometry of Tx/Rx antenna arrays and channel estimation errors.

## 1. Introduction

Full-duplex wireless communications (FDWC) for simultaneous transmission and reception (Tx/Rx) in the same frequency band [1,2,3,4,5] have attracted increasing attention recently due to the potential of doubling the spectrum efficiency. However, in order to achieve FDWC, the self-interference (SI) generated from a local transmitter to a local receiver must be mitigated for satisfactory performances [1,6], and this constitutes one of the critical challenges in FDWC.

There are basically three different approaches for SI cancellation. The first one is radio-frequency (RF) cancellation (or analog cancellation), where the RF signal to be transmitted at the Tx is exploited as a reference RF signal for SI cancellation in the Rx RF chain [1,3,4,7]. The second one is antenna cancellation, where multiple transmit (receive) antennas are carefully placed to generate two replicas with opposite phases [8,9] such that cancellation can be achieved by just adding these two replicas. The third one is digital cancellation, which is generally used together with RF or antenna cancellation to further mitigate the residual SI at baseband [1,3,4,5,8,9]. Bharadia et al. [10] showed that full-duplex (FD) radio with a combined cancellation approach is able to cancel about 110 dB SI.

In this paper, we consider a beamforming-based approach to mitigate SI for FDWC. A distinct advantage of this approach is that with beamforming cancellation, some of the conventional RF, antenna and baseband cancellation operations may be avoided, and this greatly reduces the system complexity. Beamforming cancellation is particularly meaningful for millimeter wave (mmWave) wireless communications, where large antenna arrays are typically required to compensate for the high pass loss in the mmWave frequency band [11,12,13,14,15,16,17,18,19]. Note that although frequency re-use may be easier for mmWave communication since the pass loss is high for mmWave signal, more spectrum efficiency is almost always a plus. To improve the spectral efficiency for mmWave communication may be always favored for certain scenarios, e.g., mmWave backhaul applications, where high capacity is required to support high data rate. Also, in the case of mmWave cellular with dense users, e.g., stadiums and movie theaters, FD transmission can significantly increase the multi-user capacity.

On the other hand, the FD mmWave communication does not significantly increase the system complexity. To illustrate this, let us compare the complexity of an FD-mmWave node and that of a regular mmWave node with frequency-division duplex (FDD). There are a Tx RF chain and Tx antenna array, as well as a Rx RF chain and a Rx antenna array, at both the FD-mmWave node and the regular FDD mmWave node. The only difference is that at the FD-mmWave node the Rx needs to mitigate SI, while at the regular FDD mmWave node, the Rx does not need to mitigate SI. For FD-mmWave communication, SI cancellation can be done by using the beamforming technology proposed in this paper, which only needs to control the antenna weight vectors and almost does not increase the system complexity. In brief, if beamforming technology is adopted to mitigate the SI, the complexity of an FD-mmWave node is similar to that of a regular FDD mmWave node.

However, in order to realize FD in mmWave communications, we face a joint Tx/Rx beamforming (JTR-BF) problem to maximize the Tx/Rx achievable rate. As this problem is non-convex, suboptimal solutions are expected. The existing JTR-BF schemes proposed in mmWave communications are basically infeasible, because SI was not considered in these schemes [13,20,21,22,23,24,25,26]. Although the mitigation of loopback SI [27,28] as well as the utilization of loopback SI [29,30] are considered in multiple-input multiple-output (MIMO) relays, these methods cannot be employed in our setup, as their signal models are built for relay systems, but in this paper a bi-directional FD transmission system is considered. In [31], digital beamforming to cancel SI in FDWC was studied from an experimental perspective, where the JTR-BF with SI was not analytically investigated. Although some suboptimal distributed solutions proposed for the *K*-user interference alignment (IA) problem [32,33,34,35], e.g., the max-signal-to-interference-plus-noise-ratio (Max-SINR), maximum power (Max-Power), minimum leakage (Min-Leakage), and minimum-mean-square-error (MMSE) schemes [35], may be applicable, they are all iterative solutions designed for general *K*-user IA problems. Applying them to FD mmWave communications would result in high computational complexity for large antenna arrays. Moreover, the convergence of some of them is not yet proven in the literature [35] to the best of our knowledge.

In this paper, we propose several suboptimal solutions to the JTR-BF problem for FD mmWave communications. Firstly, an iterative algorithm, which iteratively maximizes the signal power with zero-forcing (ZF) SI (ZF-Max-Power), is proposed, and its convergence is proven. Next, two closed-form solutions are derived under MMSE, ZF, and maximum-ratio transmission (MRT) criteria, namely a lower bound based MMSE solution (LB-MMSE) and ZF SI with MRT (SI-ZF-MRT), where iterations are not required. Performance evaluations show that ZF-Max-Power approaches an upper bound on the joint achievable rate, and it needs only two iterations on average to achieve the convergence with random initial points. These performances of ZF-MAx-Power are almost the same as those of the best baseline for the IA problem [32,33,34,35], namely Max-SINR. However, the convergence of Max-SINR is unproven yet [35] to the best of our knowledge, and the computational complexity of ZF-Max-Power is significantly lower than that of Max-SINR since matrix inversion is not needed. The two closed-form solutions achieve suboptimal performances to the upper bound depending on channel conditions. In addition, ZF-Max-Power and SI-ZF-MRT are robust against the geometry of Tx/Rx antenna arrays due to the operation of ZF SI, while LB-MMSE is not. ZF-Max-Power and LB-MMSE are robust against channel estimation errors, while SI-ZF-MRT is not. These results verify the feasibility of FD mmWave communication and the effectiveness of beamforming cancellation.

The rest of this paper is organized as follows. In Section 2, we introduce the system model and formulate the problem. In Section 3, we study the optimization problem, propose the ZF-Max-Power approach, and conduct the convergence analysis and complexity comparison. In Section 4, we propose the two closed-form beamforming schemes, namely LB-MMSE and SI-ZF-MRT. In Section 5, we present performance evaluations. The conclusions are drawn lastly in Section 6.

*Notation*: *a*, a, A, and A denote a scalar variable, a vector, a matrix, and a set, respectively. ${\left(\cdot \right)}^{*}$, ${\left(\cdot \right)}^{T}$ and ${\left(\cdot \right)}^{H}$ denote conjugate, transpose and conjugate transpose, respectively. In addition, $\left[x_{1},x_{2},\ldots ,x_{M}\right]$ denotes a row vector with its elements being $x_{i}$. Some other operations used in this paper are defined as follows.
E{·}Expectation operation.|x|Absolute value of scalar variable *x*.∥x∥2-norm of vector x.〈x,y〉Inner product, equals to $y^{H}x$.$x^{☆}$Optimal value of variable x.

## 2. System Model and Problem Formulation

### 2.1. System Model

**Figure 1:**
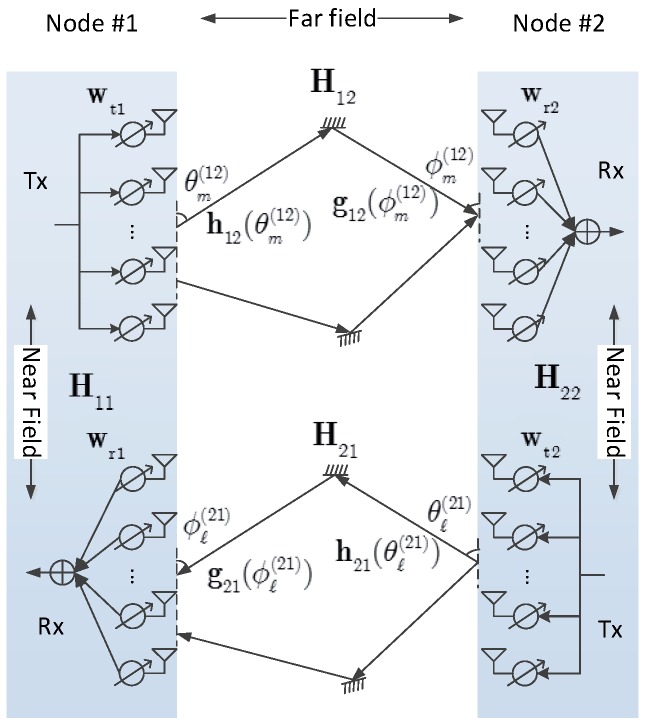
Illustration of the FD mmWave communication system.

An FD mmWave communication system consisting of two nodes, namely Node #1 and Node #2, is illustrated in Figure 1. Each node is equipped with a transmit antenna array and a receive antenna array, and supports only one data stream [21,22,36]. We denote by $n_{t1}$ and $n_{t2}$ the numbers of antenna elements of the transmit arrays at Node #1 and Node #2, respectively, while by $n_{r1}$ and $n_{r2}$ those of the receive arrays at Node #1 and Node #2, respectively. In our model, Node #1 transmits signals to Node #2 and receives signals from Node #2 simultaneously; thus both the nodes suffer from SI transmitted by the local transmitters.

It is noteworthy that although we depict separate antenna arrays for the Tx/Rx chains in Figure 1 (this structure is indeed common in FDWC [1,3,4,5,8]), the Tx/Rx chains may also share the same antenna array [1,10]. Fortunately, our signal model is suitable for both cases. Note that the SI with a shared array may be even higher than with separate arrays.

### 2.2. Channel Model

As we can see from Figure 1, there are two types of channels. The first one is the communication channel, which represents the channel for information-bearing signals exchanged between Node #1 and Node #2, i.e., $H_{12}$ and $H_{21}$, where $H_{ij}$ represents the channel from Node #*i* to Node #*j*. The other one is the SI channel. Clearly, $H_{11}$ and $H_{22}$ are the SI channels.

#### 2.2.1. Communication Channel

We first consider the model of communication channel. Since the distance between these two nodes is generally much greater than the wavelength of mmWave, the commonly used far-field channel model, which has a plane wavefront, is suitable for $H_{12}$ and $H_{21}$. According to channel measurement results for mmWave communication [13,37], mostly reflection contributes to generating multipath components (MPCs) besides the LOS component; scattering and diffraction effects are little due to the extremely short wavelength of mmWave communication. Thus, the MPCs in mmWave communication have a feature of directivity [22,23,24,38,39], i.e., different MPCs have different physical angles of departure (AoDs), i.e., $\theta_{m}^{\left(12\right)}$ and $\theta_{\ell }^{\left(21\right)}$, as well as angles of arrival (AoAs), e.g., $\phi_{m}^{\left(12\right)}$ and $\phi_{\ell }^{\left(21\right)}$, as shown in Figure 1. In general, mmWave signals have a wide band, and thus a frequency selective channel may be suitable [40]. However, with beamforming only a very small number (or even only one) of strong MPCs may be searched out to form beams between Tx and Rx. As a result, the effect of delay spread may be substantially mitigated [41]. Due to this reason, a frequency flat channel model is extensively used in mmWave communication [22,23,24,38,39], and the (narrow band) communication channels can be expressed as

(1) $$H_{12}=\sqrt{n_{t1}n_{r2}}\sum_{m=1}^{M}\alpha_{m}g_{12}\left(\phi_{m}^{\left(12\right)}\right)h_{12}^{H}\left(\theta_{m}^{\left(12\right)}\right)$$

and

(2) $$H_{21}=\sqrt{n_{t2}n_{r1}}\sum_{\ell =1}^{L}\beta_{\ell }g_{21}\left(\phi_{\ell }^{\left(21\right)}\right)h_{21}^{H}\left(\theta_{\ell }^{\left(21\right)}\right)$$

where *M* and *L* are the numbers of MPCs, $\alpha_{m}$ and $\beta_{\ell }$ are the coefficients of MPCs, $g_{12}\left(\phi_{m}^{\left(12\right)}\right)$ and $g_{21}\left(\phi_{\ell }^{\left(21\right)}\right)$ are receive steering vectors, $h_{12}\left(\theta_{m}^{\left(12\right)}\right)$ and $h_{21}\left(\theta_{\ell }^{\left(21\right)}\right)$ are transmit steering vectors of $H_{12}$ and $H_{21}$, respectively. For uniform linear arrays (ULAs) with half-wavelength spacing, these steering vectors are defined as Equation (4), and they are all functions of the corresponding steering angles. Although ULA is adopted in this paper, the developed schemes are also feasible for other types of arrays, like uniform planar array (UPA) or circular array, because different types of arrays affect only the channel matrices. For convenience, we have the following normalization:

(3) $$\sum_{m=1}^{M}E\{{\left|\alpha_{m}\right|}^{2}\}=\sum_{\ell =1}^{L}E\{{\left|\beta_{\ell }\right|}^{2}\}=1.$$

In the case of Tx/Rx sharing the same antenna array at a node, we have $n_{r1}=n_{t1}$, $n_{r2}=n_{t2}$, L=M, $\theta_{m}^{12}=\phi_{m}^{21}$, $\theta_{m}^{21}=\phi_{m}^{12}$, and $\alpha_{m}=\beta_{m}$, m=1,2,…,M, i.e., parameters of $H_{12}$ are the same as those of $H_{21}$. However, since Tx/Rx have different RF chains [1,10], the beamforming and combining vectors are basically different.

Note that for mmWave communications there may be other models. For instance, in [42] a clustered channel model was adopted, where the channel includes several clusters, and a cluster consists of many MPCs with small angle differences. Different models are suitable for different communication circumstances. As our schemes do not exploit the specific feature of the communication channel, they can be used for different models.

(4) $$\begin{array}{l} g_{12}\left(\phi_{m}^{\left(12\right)}\right)={\left[\exp\left(j\pi 0\cos(\phi_{m}^{\left(12\right)})\right),\exp\left(j\pi 1\cos(\phi_{m}^{\left(12\right)})\right),\ldots ,\exp\left(j\pi \left(n_{r2}-1\right)\cos\left(\phi_{m}^{\left(12\right)}\right)\right)\right]}^{T}/\sqrt{n_{r2}}\\ g_{21}\left(\phi_{\ell }^{\left(21\right)}\right)={\left[\exp\left(j\pi 0\cos(\phi_{\ell }^{\left(21\right)})\right),\exp\left(j\pi 1\cos(\phi_{\ell }^{\left(21\right)})\right),\ldots ,\exp\left(j\pi \left(n_{r1}-1\right)\cos\left(\phi_{\ell }^{\left(21\right)}\right)\right)\right]}^{T}/\sqrt{n_{r1}}\\ h_{12}\left(\theta_{m}^{\left(12\right)}\right)={\left[\exp\left(j\pi 0\cos(\theta_{m}^{\left(12\right)})\right),\exp\left(j\pi 1\cos(\theta_{m}^{\left(12\right)})\right),\ldots ,\exp\left(j\pi \left(n_{t1}-1\right)\cos\left(\theta_{m}^{\left(12\right)}\right)\right)\right]}^{T}/\sqrt{n_{t1}}\\ h_{21}\left(\theta_{\ell }^{\left(21\right)}\right)={\left[\exp\left(j\pi 0\cos(\theta_{\ell }^{\left(21\right)})\right),\exp\left(j\pi 1\cos(\theta_{\ell }^{\left(21\right)})\right),\ldots ,\exp\left(j\pi \left(n_{t2}-1\right)\cos\left(\theta_{\ell }^{\left(21\right)}\right)\right)\right]}^{T}/\sqrt{n_{t2}} \end{array}$$

#### 2.2.2. SI Channel

Next, we consider the strength of SI and the SI channel. Note that even in mmWave band, where the center frequency is high and the signal strength attenuates rapidly, SI may be still much more significant than the background noise, because Tx/Rx antennas locate close to each other in FD mmWave communication. For instance, if the wavelength of the carrier frequency is 1 mm, the signal bandwidth is 1 GHz, and the transmission power is 20 dBm, according to the Friis formula, the SI at a position 10 cm (100 *λ*) away from a transmit antenna is $20-20\log_{10}\left(4\pi \times 100\right)=-42$ dBm, which is much greater than a typical noise power $\sigma^{2}=10\log_{10}\left(\kappa TB\right)=10\log_{10}\left(1.38\times 10^{-23}\times 300\times 10^{9}\times 10^{3}\right)=-83.83$ dBm, where κ,T,B are the Boltzmann constant, ambient temperature and bandwidth, respectively. Hence, SI should be taken into account in FD mmWave communication.

As the distance between the transmit and receive arrays at each node is short in portable devices, the far-field range condition, i.e., $R_{0}\geq 2D^{2}/\lambda$ [43], may not hold for SI channel, where *D* is the diameter of the antenna aperture, *λ* is the wavelength of the carrier. For instance, considering a half-wavelength spaced ULA with 64 elements, the far-field range should satisfy $R_{0}\geq 2{\left(32\lambda \right)}^{2}/\lambda =2048\lambda$, which is basically too large for small-size devices like mobile phones or laptops even at the mmWave band. Thus, the SI channels may employ the near-field model, which has a spherical wavefront [43,44,45,46,47]. In such a case, the SI channels highly depend on the placement of the transmit and receive arrays as well as the circumstances.

**Figure 2:**
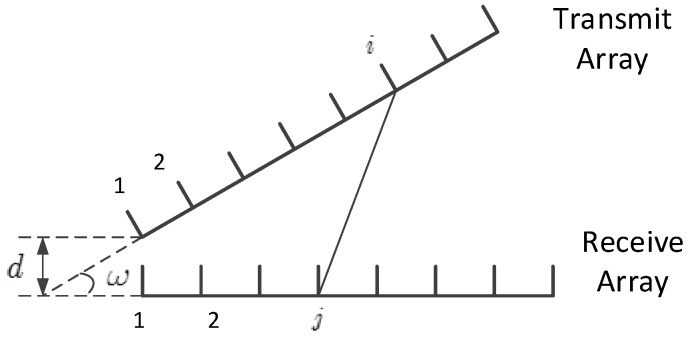
The transmit and receive antenna arrays of a node.

In this paper we consider an antenna placement as shown in Figure 2. The distance between the first elements of the two arrays is *d*, and the angle between these two ULAs is *ω*. Although this placement together with the two parameters, i.e., *d* and *ω*, cannot cover all the possible antenna placements, it allows us to perform tractable analysis. Moreover, it can reflect the effects of two typical factors of antenna placement, i.e., the distance and angle between these two arrays, on the beamforming performance. With this antenna placement, the coefficient corresponding to the *j*th row and *i*th column of $H_{11}$ or $H_{22}$ is [43,44,45,46,47].

(5) $${\left[H_{11}\;\text{or}\;H_{22}\right]}_{ij}=h_{ij}=\frac{\rho }{r_{ij}}\exp\left(-j2\pi \frac{r_{ij}}{\lambda }\right)$$

where $r_{ij}$ is the distance between the *i*-th element of the transmit array and the *j*-th element of the receive array, and *ρ* is a constant for power normalization such that $\text{tr}\left(H_{11}H_{11}^{H}\right)=n_{t1}n_{r1}$ and $\text{tr}\left(H_{22}H_{22}^{H}\right)=n_{t2}n_{r2}$. The expression for $r_{ij}$ is shown in Equation (6).

(6) $$r_{ij}=\sqrt{{\left(\frac{d}{\tan(\omega )}+\left(j-1\right)\frac{\lambda }{2}\right)}^{2}+{\left(\frac{d}{\sin(\omega )}+\left(i-1\right)\frac{\lambda }{2}\right)}^{2}-2\left(\frac{d}{\tan(\omega )}+\left(j-1\right)\frac{\lambda }{2}\right)\left(\frac{d}{\sin(\omega )}+\left(i-1\right)\frac{\lambda }{2}\right)\cos\left(\omega \right)}$$

In the case of Tx/Rx sharing the same antenna array, we have d=0 and ω=0.

The SI channel model adopted in this paper is a simplified one, which is typical [46,47] but not necessarily accurate. In practice, the SI channel can be very complicated, including signal refection, scattering and the coupling effects between adjacent antennas. Hence, it is very difficult, if not impossible, to elaborate an accurate SI channel. On the other hand, the proposed schemes can be used for arbitrary SI channels, including the completely accurate one. Hence, we adopt the simple and typical model shown in Equation (6) here, which can reflect the robustness of the proposed schemes against the geometry of the Tx/Rx antenna arrays within a node.

### 2.3. Problem Formulation

With the above system and channel models, the received signals at Node #1 and Node #2 are written as

(7) $$y_{1}=w_{r1}^{H}\left(\sqrt{\varepsilon_{21}}H_{21}w_{t2}s_{2}+\sqrt{\varepsilon_{11}}H_{11}w_{t1}s_{1}+n_{1}\right)$$

and

(8) $$y_{2}=w_{r2}^{H}\left(\sqrt{\varepsilon_{12}}H_{12}w_{t1}s_{1}+\sqrt{\varepsilon_{22}}H_{22}w_{t2}s_{2}+n_{2}\right)$$

respectively, where $s_{1}$ and $s_{2}$ are the transmitted symbols with unit power at Node #1 and Node #2, respectively, $\varepsilon_{21}$ and $\varepsilon_{12}$ are the average powers of the desired received signals, $\varepsilon_{11}$ and $\varepsilon_{22}$ are the average powers of the SI, $n_{1}$ and $n_{2}$ are the Gaussian white noise vectors with $E\left\{n_{1}n_{1}^{H}\right\}=I_{n_{r1}}$ and $E\left\{n_{2}n_{2}^{H}\right\}=I_{n_{r2}}$, respectively, $w_{t1}$ and $w_{t2}$ are the transmit antenna weight vectors (AWVs), and $w_{r1}$ and $w_{r2}$ are the receive AWVs. The two-norms of all these AWVs are normalized to 1.

With the transmit and receive AWVs, the joint achievable rate (JAR) can be expressed as

(9) $$R=\log_{2}\left(1+\frac{\varepsilon_{21}{\left|w_{r1}^{H}H_{21}w_{t2}\right|}^{2}}{1+\varepsilon_{11}{\left|w_{r1}^{H}H_{11}w_{t1}\right|}^{2}}\right)+\log_{2}\left(1+\frac{\varepsilon_{12}{\left|w_{r2}^{H}H_{12}w_{t1}\right|}^{2}}{1+\varepsilon_{22}{\left|w_{r2}^{H}H_{22}w_{t2}\right|}^{2}}\right)$$

where $\varepsilon_{11}{\left|w_{r1}^{H}H_{11}w_{t1}\right|}^{2}$ and $\varepsilon_{22}{\left|w_{r2}^{H}H_{22}w_{t2}\right|}^{2}$ are the average powers of SI at Nodes #1 and #2, respectively. The JTR-BF problem is formulated as

(10) $$\begin{array}{cc} \underset{w_{t1},w_{r1},w_{t2},w_{r2}}{\text{maximize}} & R\\ \text{subject}\;\text{to} & {∥w_{t1}∥}^{2}={∥w_{r1}∥}^{2}=1\\ & {∥w_{t2}∥}^{2}={∥w_{r2}∥}^{2}=1 \end{array}$$

As the main purpose of this paper is to investigate the feasibility of FD mmWave communications and evaluate the performance of beamforming cancellation, the channel matrices and powers in Equation (10) are assumed known *a-priori*. In practice, these parameters can be estimated provided that the channel does not change too fast. For instance, as an mmWave communication channel has the feature of directivity and is sparse in the angle domain, schemes like AoD/AoA estimation [48], beam searching [26,39,49], iterative training [22,50], and even compressed sensing [51,52] can be adopted for the communication channel estimation. The estimation of SI channel can be more straightforward, e.g., one can estimate a scalar channel coefficient between a single Tx/Rx antenna pair (i.e., the *i*-th transmit antenna and the *j*-th receive antenna) once a time. After $n_{ri}n_{ti}$ (i=1,2) measurements, the SI channel matrix can be estimated. Although $n_{ri}n_{ti}$ is large in mmWave communication, each measurement may require only one symbol thanks to the high strength of SI, rather than a long training sequence like those in [22,26,39,49,50,51]. Thus, the time cost of the SI channel estimation is yet affordable.

In addition, in our model both amplitudes and phases of the AWVs are controllable. In fact, a constant-amplitude (CA) array with only phases controllable has lower complexity [22,26,39,49,51]. An *N*-element CA array needs *N* phase shifters, which are not difficult to implement. In contrast, an *N*-element array with both amplitudes and phases controllable requires *N* phase shifters and *N* variable gain amplifier. Hence, a CA array has a lower complexity than an amplitude-and-phase-controllable array, especially when *N* is large, and thus is favored in mmWave communications, where *N* is large in general. However, the array structure without the CA constraint cannot be definitely ruled out, because it also arouses particular attention in both algorithm design [23,24,50,53,54] and implementation [55]. Moreover, the performance achieved with non-CA-constraint arrays can be seen as a bound achieved with the CA arrays, if the same scheme is adopted. Therefore, in this paper we adopt the arrays without the CA constraint to investigate the feasibility of FD mmWave communication and evaluate the beamforming performance, letting the problem with CA constraint be a further work.

## 3. The ZF-Max-Power Approach

It is clear that the JAR in Equation (10) is not a concave function, and the equality constraints are not affine. Thus, Equation (10) is not a convex/concave problem, and its globally optimal solution is hard to find. Consequently, we first give an upper bound JAR, and then propose the ZF-Max-Power approach in this section.

### 3.1. Upper Bound of the JAR

According to Equation (9), since $\varepsilon_{11}{\left|w_{r1}^{H}H_{11}w_{t1}\right|}^{2}\geq 0$ and $\varepsilon_{22}{\left|w_{r2}^{H}H_{22}w_{t2}\right|}^{2}\geq 0$, an *upper bound function* on the JAR, denoted by $R_{\text{ub}}$, in the presence of SI can be easily obtained as

(11) $$R\leq \log_{2}\left(1+\varepsilon_{21}{\left|w_{r1}^{H}H_{21}w_{t2}\right|}^{2}\right)+\log_{2}\left(1+\varepsilon_{12}{\left|w_{r2}^{H}H_{12}w_{t1}\right|}^{2}\right)≜R_{\text{ub}}$$

The corresponding optimal AWVs for $R_{\text{ub}}$ are

(12) $$\begin{array}{c} w_{t1}=\text{RpSingVect}\left(H_{12}\right),\;w_{t2}=\text{RpSingVect}\left(H_{21}\right)\\ w_{r1}=\text{LpSingVect}\left(H_{21}\right),\;w_{r2}=\text{LpSingVect}\left(H_{11}\right) \end{array}$$

where LpSingVect(X) and RpSingVect(X) represent the left and right principal singular vectors of X, respectively. Thus, we have

(13) $$R\leq R_{\text{ub}}\leq R_{\text{ub}}^{☆}<\infty$$

where $R_{\text{ub}}^{☆}$ is the maximum of the upper bound $R_{\text{ub}}$, and thus it is an upper bound on *R*.

### 3.2. The ZF-Max-Power Approach

As there are multiple coupled variables in Equation (10), we consider using the alternating optimization (AO) approach [56] to obtain a suboptimal solution of Equation (10). The basic idea of AO is to alternately optimize a few parameters, and assume the other parameters fixed and known [56]. In each round, a sub-problem with a few parameters are formulated and solved. Generally, this approach requires that the optimal solution to each sub-problem can be found. However, for the problem in Equation (10), the AO approach cannot be directly used, because, as we can see, even given $w_{r1}$ and $w_{r2}$, optimal $w_{t1}$ and $w_{t2}$ still cannot be easily found.

To make the AO approach feasible, we propose the ZF-Max-Power scheme in this paper. The motivation of this scheme is as follows. Since the SI is usually significant in FD mmWave communication, we can force the SI to zero and maximize the signal power. In particular, we add a constraint that the SI is completely mitigated. In such a case, we have $R=R_{\text{ub}}$, and the problem becomes

(14) $$\begin{array}{cc} \underset{w_{t1},w_{r1},w_{t2},w_{r2}}{\text{maximize}} & R_{\text{ub}}\\ \text{subject}\;\text{to} & {∥w_{t1}∥}^{2}={∥w_{r1}∥}^{2}=1\\ & {∥w_{t2}∥}^{2}={∥w_{r2}∥}^{2}=1\\ & w_{r1}^{H}H_{11}w_{t1}=w_{r2}^{H}H_{22}w_{t2}=0 \end{array}$$

Since we have added a new constraint to the original problem in Equation (10), the solution of the new problem in Equation (14) is suboptimal to the original problem. To solve the new problem in Equation (14), we need the following result.

**Lemma** **1.**  *Given an arbitrary set of linearly independent vectors* ${\left\{a_{i}\in C^{L\times 1}\right\}}_{i=1,2,\ldots ,N;\;N<L}$ *and an arbitrary vector* $a\in C^{L\times 1}$*, the vector* $b\in C^{L\times 1}$ *that maximizes* ${\left|\langle b,a\rangle \right|}^{2}$ *with the constraint that* $\langle b,a_{i}\rangle {=0|}_{i=1,2,\ldots ,N}$ *and* ∥b∥=1 *is (before normalization)* $b=a-\sum_{i=1}^{N}\langle a,b_{i}\rangle b_{i}$*, where* $b_{1}=a_{1}/∥a_{1}∥$ *and*

(15) $$b_{i}=\frac{a_{i}-\sum_{j=1}^{i-1}\langle a_{i},b_{j}\rangle b_{j}}{∥a_{i}-\sum_{j=1}^{i-1}\langle a_{i},b_{j}\rangle b_{j}∥},\;i>1$$

**Proof.**  See Appendix A.  ☐

Specifically, when N=1 the optimal b in Lemma 1 before normalization is

(16) $$b^{☆}=a-\langle a,\frac{a_{1}}{∥a_{1}∥}\rangle \frac{a_{1}}{∥a_{1}∥}$$

and when N=2 the optimal b in Lemma 1 before normalization is

(17) $$b^{☆}=a-\langle a,\frac{a_{1}}{∥a_{1}∥}\rangle \frac{a_{1}}{∥a_{1}∥}-\langle a,b_{2}\rangle b_{2}$$

where

(18) $$b_{2}=\frac{a_{2}-\langle a_{2},\frac{a_{1}}{∥a_{1}∥}\rangle \frac{a_{1}}{∥a_{1}∥}}{∥a_{2}-\langle a_{2},\frac{a_{1}}{∥a_{1}∥}\rangle \frac{a_{1}}{∥a_{1}∥}∥}$$

Let us go back to the ZF-Max-Power scheme. According to Equation (16), given fixed $w_{t1}$ and $w_{t2}$, the optimal $w_{r1}$ and $w_{r2}$ for Equation (14) (before normalization) are

(19) $$w_{r1}=H_{21}w_{t2}-\langle H_{21}w_{t2},\frac{H_{11}w_{t1}}{∥H_{11}w_{t1}∥}\rangle \frac{H_{11}w_{t1}}{∥H_{11}w_{t1}∥}$$

and

(20) $$w_{r2}=H_{12}w_{t1}-\langle H_{12}w_{t1},\frac{H_{22}w_{t2}}{∥H_{22}w_{t2}∥}\rangle \frac{H_{22}w_{t2}}{∥H_{22}w_{t2}∥}$$

respectively.

Similarly, given fixed $w_{r1}$ and $w_{r2}$, the optimal $w_{t1}$ and $w_{t2}$ for Equation (14) (before normalization) are

(21) $$w_{t1}=H_{12}^{H}w_{r2}-\langle H_{12}^{H}w_{r2},\frac{H_{11}^{H}w_{r1}}{∥H_{11}^{H}w_{r1}∥}\rangle \frac{H_{11}^{H}w_{r1}}{∥H_{11}^{H}w_{r1}∥}$$

and

(22) $$w_{t2}=H_{21}^{H}w_{r1}-\langle H_{21}^{H}w_{r1},\frac{H_{22}^{H}w_{r2}}{∥H_{22}^{H}w_{r2}∥}\rangle \frac{H_{22}^{H}w_{r2}}{∥H_{22}^{H}w_{r2}∥}$$

respectively. Finally, the ZF-Max-Power scheme can be summarized as in Algorithm 1.
**Algorithm 1** The ZF-Max-Power Scheme.**(1) Initialize:**
Initialize the transmit AWVs as $w_{t1}=\text{RpSingVect}\left(H_{12}\right),\;w_{t2}=\text{RpSingVect}\left(H_{21}\right)$.
**(2) Iteration:**
Iterate the following process *ρ* times, then stop.Compute $w_{r1}$ and $w_{r2}$ according to Equations (19) and (20), respectively; then normalize them.Compute $w_{t1}$ and $w_{t2}$ according to Equations (21) and (22), respectively; then normalize them.
**(3) Result:**
$w_{t1}$ and $w_{t2}$ are the transmit AWVs, and $w_{r1}$ and $w_{r2}$ are the receive AWVs.



It is noted that there are various stopping rules for the ZF-Max-Power scheme. A simple one is to stop after a certain number of iterations, e.g., *ρ* iterations used in Algorithm 1. Another one is to compute $R_{\text{ub}}$ after the *n*-th iteration and get $R_{\text{ub}}^{\left(n\right)}$ according to Equation (9). When $R_{\text{ub}}^{\left(n\right)}/R_{\text{ub}}^{\left(n-1\right)}<\mu$, stop the iteration, where *μ* is a predefined threshold slightly greater than 1, e.g., μ=1.05.

### 3.3. Convergence Analysis and Complexity Comparison

To prove the convergence of the ZF-Max-Power scheme, we need the following theorem.

**Theorem** **1.**  *Let* $R^{\left(n\right)}$ *denote the value of R after the n-th iteration. Then* $\left\{R^{\left(n\right)}|n=1,2,\ldots \right\}$ *is a non-descending sequence, i.e.,* $R^{\left(n+1\right)}\geq R^{\left(n\right)}$.

**Proof.**  See Appendix B.  ☐

According to Equation (13), $R\leq R_{\text{ub}}^{☆}<\infty$. Thus, $\left\{R^{\left(n\right)}|n=1,2,\ldots \right\}$ converges to a suboptimal value, which guarantees the convergence of ZF-Max-Power.

On the other hand, the computational complexity of ZF-Max-Power is much lower than that of Max-SINR [35]. For simplicity, suppose $n_{t1}=n_{t2}=n_{r1}=n_{r2}=N$. According to Algorithm 1, in each iteration the main complexity of ZF-Max-Power lies in multiplications between a channel matrix and an AWV, as shown in Equations (19)–(22). Hence, the computational complexity of ZF-Max-Power is roughly $O(N^{2})$, because there are about $N^{2}$ scalar multiplications for a multiplication of a channel matrix and an AWV. In contrast, according to [35] matrix inversion is required in each iteration of Max-SINR. Hence, Max-SINR has a computational complexity of $O(N^{3})$, which is significantly higher than that of ZF-Max-Power, especially in FD mmWave communication where *N* is large.

## 4. Closed-Form Solutions

In this section, we consider different criteria for the JTR-BF problem that provide us with closed-form solutions.

### 4.1. LB-MMSE

It is natural to perform receive and transmit beamforming separately when considering closed-form solutions. According to Equation (9), an optimal solution can be achieved for receive beamforming by using the MMSE approach which is equivalent to maximizing the SINR. However, for transmit beamforming, the optimal solution is hard to find. Thus, we propose to optimize the lower bound on the JAR with MMSE, and the method is referred to as the lower bound MMSE (LB-MMSE) approach.

According to Equation (9), we have

(23) $$\begin{array}{cc} R & \geq \log_{2}\left(\frac{\varepsilon_{21}{\left|w_{r1}^{H}H_{21}w_{t2}\right|}^{2}}{w_{r1}^{H}w_{r1}+\varepsilon_{11}{\left|w_{r1}^{H}H_{11}w_{t1}\right|}^{2}}\frac{\varepsilon_{12}{\left|w_{r2}^{H}H_{12}w_{t1}\right|}^{2}}{w_{r2}^{H}w_{r2}+\varepsilon_{22}{\left|w_{r2}^{H}H_{22}w_{t2}\right|}^{2}}\right)\\ & =\log_{2}\left(\frac{\varepsilon_{21}w_{r1}^{H}H_{21}w_{t2}w_{t2}^{H}H_{21}^{H}w_{r1}}{w_{r1}^{H}\left(I+\varepsilon_{11}H_{11}w_{t1}w_{t1}^{H}H_{11}^{H}\right)w_{r1}}\frac{\varepsilon_{12}w_{r2}^{H}H_{12}w_{t1}w_{t1}^{H}H_{12}^{H}w_{r2}}{w_{r2}^{H}\left(I+\varepsilon_{22}H_{22}w_{t2}w_{t2}^{H}H_{22}^{H}\right)w_{r2}}\right)≜R_{1} \end{array}$$

First, we find $w_{r1}$ and $w_{r2}$ to maximize the lower bound $R_{1}$ by temporally treating $w_{t1}$ and $w_{t2}$ as fixed parameters. This subproblem is actually to maximize SINR (or minimize MSE) at the two nodes. According to Lemma 2 in Appendix C, the optimal $w_{r1}$ and $w_{r2}$ (before normalization) can be respectively found as

(24) $$\begin{array}{c} w_{r1}={\left(I+\varepsilon_{11}H_{11}w_{t1}w_{t1}^{H}H_{11}^{H}\right)}^{-1}H_{21}w_{t2}\\ w_{r2}={\left(I+\varepsilon_{22}H_{22}w_{t2}w_{t2}^{H}H_{22}^{H}\right)}^{-1}H_{12}w_{t1} \end{array}$$

With these two receive AWVs, we further have Equation (25), where inequalities (a) and (b) are based on Lemmas 3 and 4, respectively, in Appendix C.

(25) $$\begin{array}{cc} R_{1} & =\log_{2}\left(\varepsilon_{12}\varepsilon_{21}\left(w_{t2}^{H}H_{21}^{H}{\left(I+\varepsilon_{11}H_{11}w_{t1}w_{t1}^{H}H_{11}^{H}\right)}^{-1}H_{21}w_{t2}\right)\left(w_{t1}^{H}H_{12}^{H}{\left(I+\varepsilon_{22}H_{22}w_{t2}w_{t2}^{H}H_{22}^{H}\right)}^{-1}H_{12}w_{t1}\right)\right)\\ & \overset{\left(a\right)}{\geq }\log_{2}\left(\varepsilon_{12}\varepsilon_{21}\frac{{\left(w_{t2}^{H}H_{21}^{H}H_{21}w_{t2}\right)}^{2}}{w_{t2}^{H}H_{21}^{H}\left(I+\varepsilon_{11}H_{11}w_{t1}w_{t1}^{H}H_{11}^{H}\right)H_{21}w_{t2}}\frac{{\left(w_{t1}^{H}H_{12}^{H}H_{12}w_{t1}\right)}^{2}}{w_{t1}^{H}H_{12}^{H}\left(I+\varepsilon_{22}H_{22}w_{t2}w_{t2}^{H}H_{22}^{H}\right)H_{12}w_{t1}}\right)\\ & \overset{\left(b\right)}{\geq }\log_{2}\left(\varepsilon_{12}\varepsilon_{21}\frac{w_{t2}^{H}H_{21}^{H}H_{21}w_{t2}}{w_{t1}^{H}\left(I+\varepsilon_{11}H_{11}^{H}H_{11}\right)w_{t1}}\frac{w_{t1}^{H}H_{12}^{H}H_{12}w_{t1}}{w_{t2}^{H}\left(I+\varepsilon_{22}H_{22}^{H}H_{22}\right)w_{t2}}\right)\\ & =\log_{2}\left(\varepsilon_{12}\varepsilon_{21}\frac{w_{t2}^{H}H_{21}^{H}H_{21}w_{t2}}{w_{t2}^{H}\left(I+\varepsilon_{22}H_{22}^{H}H_{22}\right)w_{t2}}\frac{w_{t1}^{H}H_{12}^{H}H_{12}w_{t1}}{w_{t1}^{H}\left(I+\varepsilon_{11}H_{11}^{H}H_{11}\right)w_{t1}}\right)≜R_{2} \end{array}$$

Next, let us find $w_{t1}$ and $w_{t2}$ to maximize the lower bound $R_{2}$. This subproblem is equivalent to the following two optimization problems:

(26) $$\underset{w_{t1}}{\arg}\;\max\;\frac{w_{t1}^{H}H_{12}^{H}H_{12}w_{t1}}{w_{t1}^{H}\left(I+\varepsilon_{11}H_{11}^{H}H_{11}\right)w_{t1}}$$

and

(27) $$\underset{w_{t2}}{\arg}\;\max\;\frac{w_{t2}^{H}H_{21}^{H}H_{21}w_{t2}}{w_{t2}^{H}\left(I+\varepsilon_{22}H_{22}^{H}H_{22}\right)w_{t2}}$$

These are generalized Rayleigh quotient problems, and the optimal transmit AWVs (before normalization) are

(28) $$w_{t1}=\text{pEigVect}\left({\left(I+\varepsilon_{11}H_{11}^{H}H_{11}\right)}^{-1}H_{12}^{H}H_{12}\right)$$

and

(29) $$w_{t2}=\text{pEigVect}\left({\left(I+\varepsilon_{22}H_{22}^{H}H_{22}\right)}^{-1}H_{21}^{H}H_{21}\right)$$

respectively, where pEigVect(X) denotes the principal eigenvector of X.

In brief, by exploiting the proposed LB-MMSE, the transmit AWVs are computed as Equations (28) and (29), respectively. Based on the transmit AWVs, the receive AWVs (before normalization) are found as Equation (24).

### 4.2. SI-ZF-MRT

In this scheme, we first consider transmit beamforming by adopting MRT, and then use ZF to suppress the SI for receive beamforming. This approach is referred to as SI-ZF-MRT.

By exploiting MRT for transmit beamforming, we have

(30) $$w_{t1}=H_{12}^{H}w_{r2};\;w_{t2}=H_{21}^{H}w_{r1}$$

To suppress the SI, we have

(31) $$w_{r1}^{H}H_{11}H_{12}^{H}w_{r2}=0=w_{r2}^{H}H_{22}H_{21}^{H}w_{r1}$$

There are many solutions of $w_{r1}$ and $w_{r2}$ for Equation (31). Among those, we want to find the receive AWVs to maximize JAR while satisfying Equation (31). With Equations (30) and (31), the JAR becomes

(32) $$\begin{array}{cc} R & =\log_{2}\left(1+\varepsilon_{21}{\left|w_{r1}^{H}H_{21}H_{21}^{H}w_{r1}\right|}^{2}\right)+\log_{2}\left(1+\varepsilon_{12}{\left|w_{r2}^{H}H_{12}H_{12}^{H}w_{r2}\right|}^{2}\right) \end{array}$$

There are two options to design $w_{r1}$ and $w_{r2}$. One is to first derive $w_{r1}$ that maximizes Equation (32) without considering the constraint Equation (31); then derive $w_{r2}$ to optimize Equation (32) with Equation (31) satisfied. With this option, we have

(33) $$w_{r1}=\text{pEigVect}\left(H_{21}H_{21}^{H}\right)$$

and

(34) $$w_{r2}=a-\langle a,\frac{a_{1}}{∥a_{1}∥}\rangle \frac{a_{1}}{∥a_{1}∥}-\langle a,b_{2}\rangle b_{2}$$

where $a=\text{pEigVect}\left(H_{12}H_{12}^{H}\right)$, $a_{1}=H_{12}H_{11}^{H}w_{r1}$, and

(35) $$b_{2}=\frac{H_{22}H_{21}^{H}w_{r1}-\langle H_{22}H_{21}^{H}w_{r1},\frac{a_{1}}{∥a_{1}∥}\rangle \frac{a_{1}}{∥a_{1}∥}}{∥H_{22}H_{21}^{H}w_{r1}-\langle H_{22}H_{21}^{H}w_{r1},\frac{a_{1}}{∥a_{1}∥}\rangle \frac{a_{1}}{∥a_{1}∥}∥}$$

The other one is similar to the first one but with the positions of $w_{r1}$ and $w_{r2}$ exchanged. With this option, we have

(36) $$w_{r2}=\text{pEigVect}\left(H_{12}H_{12}^{H}\right)$$

and

(37) $$w_{r1}=a-\langle a,\frac{a_{1}}{∥a_{1}∥}\rangle \frac{a_{1}}{∥a_{1}∥}-\langle a,b_{2}\rangle b_{2}$$

where $a=\text{pEigVect}\left(H_{21}H_{21}^{H}\right)$, $a_{1}=H_{11}H_{12}^{H}w_{r2}$, and

(38) $$b_{2}=\frac{H_{21}H_{22}^{H}w_{r2}-\langle H_{21}H_{22}^{H}w_{r2},\frac{a_{1}}{∥a_{1}∥}\rangle \frac{a_{1}}{∥a_{1}∥}}{∥H_{21}H_{22}^{H}w_{r2}-\langle H_{21}H_{22}^{H}w_{r2},\frac{a_{1}}{∥a_{1}∥}\rangle \frac{a_{1}}{∥a_{1}∥}∥}$$

In brief, for SI-ZF-MRT, the two options are (i) to find the receive AWVs according to Equations (33) and (34), and then obtain the transmit AWVs according to Equation (30); (ii) to find the receive AWVs according to Equations(36) and (37), and then obtain the transmit AWVs according to Equation (30). Therefore, the one which has a higher JAR can be selected as the solution for SI-ZF-MRT.

Similar to SI-ZF-MRT, SI-ZF-maximum-ratio combining (MRC) is also applicable to the joint beamforming problem. With SI-ZF-MRC, receive beamforming is firstly performed by using MRC. Afterwards, transmit beamforming is carried out to maximize the JAR with the SI forced to zero. Since SI-ZF-MRC is similar to SI-ZF-MRT in formulation and performance, we do not present the details here.

### 4.3. Steering Beamforming

The conventional SBF in mmWave communication can also be introduced here to compare with the alternatives. SBF does not require full channel information. Instead, it only requires the knowledge of the transmit and steering vectors for the most significant MPC of the communication channel, and does not consider the SI. Suppose the *m*-th and *ℓ*-th multipath components are the most significant ones from Node #1 to Node #2 and from Node #2 to Node #1, respectively. By using SBF, the AWVs become

(39) $$\begin{array}{c} w_{t2}=h_{21}\left(\theta_{\ell }^{\left(21\right)}\right),\;w_{t1}=h_{12}\left(\theta_{m}^{\left(12\right)}\right)\\ w_{r1}=g_{21}\left(\phi_{\ell }^{\left(21\right)}\right),\;w_{r2}=g_{12}\left(\phi_{m}^{\left(12\right)}\right) \end{array}$$

By comparing the performance of SBF with those of the proposed schemes, we can see whether or not it is infeasible not to consider the SI in FD mmWave communication, and how much performance degradation it causes if we do not consider the SI in beamforming.

## 5. Simulation Results

In this section, we present the performances of all the involved schemes through numerical simulations, where the communication channel model and the SI channel model introduced in Section 2 are adopted. In all the evaluations, the near-field SI channels are deterministic, and are decided by Equation (5); while the far-field signal channels are random. Both LOS and non-LOS (NLOS) channels are considered for the communication channels. For NLOS channel, the transmit and receive steering angles are randomly generated within [0,2π), and the coefficients obey circularly symmetric complex Gaussian distribution with the same average power. For LOS channel, the LOS component has a fixed coefficient and fixed transmit and receive steering angles, while the other NLOS components have random steering angles and coefficients with average power 15 dB lower than the LOS component. The total power of a generated channel obeys Equation (3), and the total number of MPCs is 3 (We’ve also simulated with other numbers of MPCs, and similar results were obtained.). For each curve in all the figures in this section, we have generated 1000 realizations with the LOS or NLOS channel models, and computed the average JAR based on these realizations. Moreover, we have considered both types of array settings in the evaluations, namely Tx/Rx have separate antenna arrays and the same antenna array at a node. In all the simulations, $n_{t1}=n_{r1}=n_{t2}=n_{r2}=32$.

**Figure 3:**
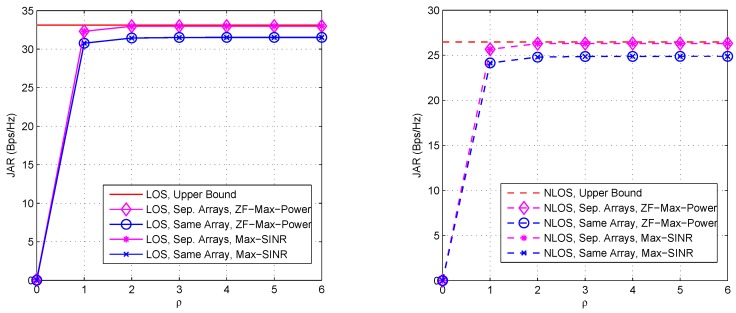
JAR and convergence performances of ZF-Max-Power with random initial transmit AWVs (**Left**: LOS channel, **Right**: NLOS channel). $\varepsilon_{11}=\varepsilon_{22}=40$ dB. For LOS channel, $\varepsilon_{12}=\varepsilon_{21}=20$ dB, while for NLOS channel, $\varepsilon_{12}=\varepsilon_{21}=10$ dB. For the case of separate arrays, d/λ=1 and ω=π/6 rad, while for the case of sharing the same array, d/λ=0 and ω=0 rad.

Firstly, we consider the JAR and convergence performances of the ZF-Max-Power scheme with random initial transmit AWVs, which are shown in Figure 3 with relevant parameters listed in the caption. Both cases of separate arrays and the same array are included under LOS/NLOS channels. As ZF-Max-Power is an iterative method, we compare it with Max-SINR, which achieves the best performance within the typical solutions for the IA problem [35]. It is observed that ZF-Max-Power achieves a suboptimal performance close to the upper bound after convergence, under both LOS and NLOS channels, especially with separate arrays. The slight superiority of the case with separate arrays is due to that the different channel parameters of $H_{12}$ and $H_{21}$ provide more degrees of spatial freedoms for beamforming than the case of the same array, where the channel parameters are the same. Moreover, the convergence speed of ZF-Max-Power is fast in all the cases. Basically, only two iterations are required to achieve convergence. Interestingly, under the considered scenario, ZF-Max-Power achieves almost the same JAR and convergence performances as Max-SINR. However, it is noteworthy that ZF-Max-Power is a centralized iterative approach, where the iteration is performed at a certain node, and does not exploit channel reciprocity. By contrast, Max-SINR is a distributed iterative approach, where the iteration is performed at all distributed nodes by exploiting channel reciprocity. As a consequence, the convergence of ZF-Max-Power can be proven, while that of Max-SINR is still unproven in the literature [35] to the best of our knowledge. Moreover, ZF-Max-Power has a significantly lower computational complexity than Max-SINR.

**Figure 4:**
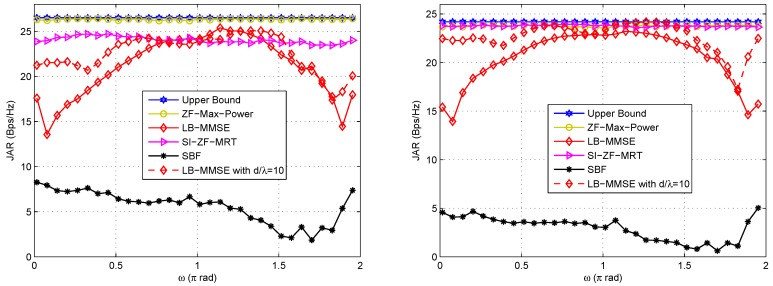
JAR performance of the involved schemes with respect to varying *ω* under LOS (**Left**) and NLOS (**Right**) channels in the case of separate arrays. $\varepsilon_{11}=\varepsilon_{22}=40$ dB, $\varepsilon_{12}=\varepsilon_{21}=10$ dB, d/λ=2. For LB-MMSE, the JAR with d/λ=10 is also plotted.

**Figure 5:**
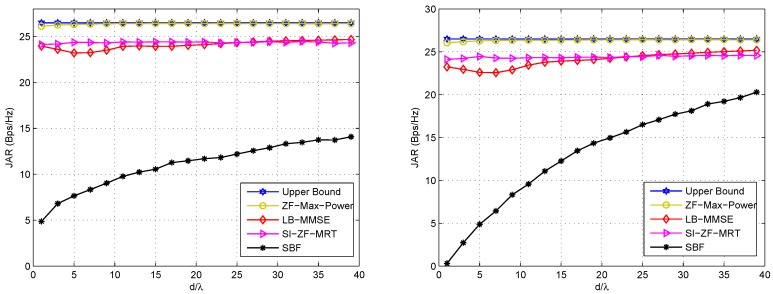
JAR performance of the involved schemes with respect to varying *d* under LOS channel in the case of separate arrays. ω=π rad. In the (**Left**) hand figure SI is assumed fixed, i.e., $\varepsilon_{11}=\varepsilon_{22}=40$ dB, $\varepsilon_{12}=\varepsilon_{21}=10$ dB; while in the (**Right**) hand figure SI varies with d/λ, i.e., $\varepsilon_{11}=\varepsilon_{22}=60-20\log_{10}\left(d/\lambda \right)$ dB, $\varepsilon_{12}=\varepsilon_{21}=10$ dB.

Next, let us see the JAR performances of the proposed schemes with respect to varying *ω*, *d*, i.e., the geometry of the arrays, when separate arrays are exploited at a node. Figure 4 shows the JAR performances with respect to varying *ω* under LOS and NLOS channels, respectively, where SI is fixed. The left hand side figure of Figure 5 shows the JAR performances with respect to *d* under LOS channel. Similar results can be observed under NLOS channel. As we can see, SI is assumed fixed in the left hand side figure of Figure 5, which may be not practically reasonable, because in practice *d* significantly affects SI. However, in order to adequately evaluate the effects of *d* on the JAR performance, we assume a fixed SI in the left hand side figure of Figure 5. For rigorousness, we also adopt varying SI in the right hand side figure of Figure 5, which is in accordance with the practice, where SI deteriorates with $d^{2}$. It is noteworthy that the strength of SI is typically much higher than that of the desired signal, because SI comes from the local transmitter at the same node, while the designed signal comes from the remote transmitter at the other node. Relevant parameters for these figures are listed in the corresponding captions.

By comparing these figures with each other, it can be observed that:

(i) ZF-Max-Power is robust against *ω*, *d* and SI, and approaches the upper bound in all these cases. This is because ZF-Max-Power not only forces SI to zero, but also iteratively maximizes signal power. Thus, it achieves compelling performance that is insensitive to the geometry of the Tx/Rx arrays and SI.
(ii) SI-ZF-MRT is also robust against *ω*, *d* and SI, thanks to its zero-forcing filtering to SI. In addition, it also achieves an acceptable performance, which is close to the upper bound. It is noted that the JAR gap between SI-ZF-MRT and the upper bound is greater under LOS channel than that under NLOS channel. This phenomenon can be explained by referring to Equation (34), where $w_{r2}$ is in fact set within an $\left(n_{r2}-2\right)$-dimension subspace, due to the two zero forcing equations shown in Equation (31). Clearly if a in Equation (34), which represents the dimension with the largest power of the channel, has less energy projected on the $\left(n_{r2}-2\right)$-dimension subspace, the JAR performance will be poorer. Under LOS channel, the majority of the channel energy concentrates on a single path, or a single dimension. Once this dimension has a small projection on the subspace, the performance will be poor. In contrast, under NLOS channel the channel energy evenly disperses on multiple paths. Only when all of these paths have a small projection on the subspace, the performance will be poor. In other words, the probability of a poor performance is lower under NLOS channel than that under LOS channel. Hence, on average the JAR gap between SI-ZF-MRT and the upper bound is greater under LOS channel than that under NLOS channel.
(iii) LB-MMSE is sensitive to *ω* and *d*. From Figure 4 we observe that the performance of LB-MMSE fluctuates as *ω* changes, and the fluctuation is different for different *d*. From Figure 5 we observe that the performance of LB-MMSE has a ∪-shape as *d* increases, but behaves stable when *d* is large. To understand these, we need to go back to Equations (28) and (29). From these two equations we can see that the transmit AWVs are decided to maximize the SINR rather than minimize SI based on the local information. Taking Equation (28) for illustration, since usually $\varepsilon_{11}$ is big, when $H_{11}$ has a low rank, the eigenvector of $H_{12}^{H}H_{12}$ has a high probability to locate within the null space of $H_{11}$. In such a case, a high signal power can be achieved while little SI locates within the signal subspace; thus good performance is achieved. Note that this statement is just for illustration. In practice, $H_{11}$ is generally with full rank except when ω=0 or *π*. However, when most energy of $H_{11}$ locates at a low-dimensional subspace, the situation will be similar to the statement that $H_{11}$ has a low rank. In comparison, when $H_{11}$ has a high or even full rank, SI will almost unavoidably locate within the signal subspace and affects the received SINR, and thus the performance will be poor. When *d* is small, the energy dispersion of $H_{11}$ is sensitive to *ω* and *d* according to the SI channel model, and thus the JAR performance is also sensitive to *ω* and *d*. However, when *d* is large, the SI channel almost reduces to a directional channel with rank 1, and thus SI has a low probability to locate within the signal subspace. In such a case, LB-MMSE can stably achieve a near-optimal performance.
(iv) SBF is also sensitive to *ω*, *d* and SI. This is because SBF does not even consider SI in the beamforming design. Meanwhile, from Figure 5 it is found that SBF becomes improved as *d* increases. In the right hand side of Figure 5 the improving speed of SBF is faster than that in Figure 5, because SI is reduced as *d* increases. This phenomenon suggests that when the near-field SI channel gradually reduces to a directional channel, the conventional beamforming schemes that to simply steer towards each other may also achieve good performance, because usually the communication channel and SI channel have difference steering angles. However, in practical FD mmWave communication where *d* is generally small, the SI channel does not have the feature of directivity; thus SBF is much poorer than the other candidates, and the performance of SBF does not show monotonicity with *ω*, as shown in Figure 4. Thus, SBF may not be a good choice for FD mmWave communication, where SI must be taken into account.

**Figure 6:**
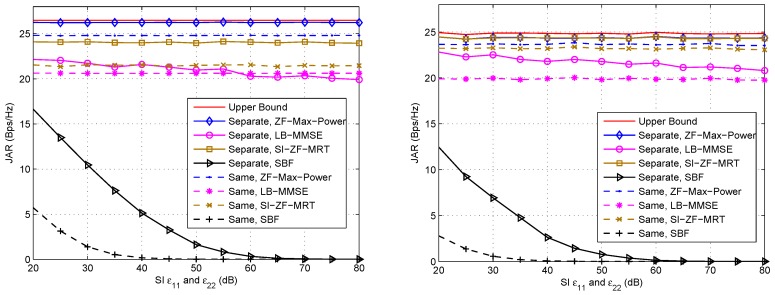
JAR comparison between different array settings (separate arrays versus the same array) under LOS (**Left**) and NLOS (**Right**) channels with varying SI. $\varepsilon_{12}=\varepsilon_{21}=10$ dB. For the case of separate arrays, ω=0.6π rad, d/λ=1.

Then, we compare the JAR performances of the discussed schemes with separate arrays and the same array. Figure 6 shows the comparison results with respect to SI under LOS and NLOS channels, respectively, where relevant parameters are listed in the captions. From these two figures we observe that the schemes with separate arrays basically achieve better performance than those with the same array. This advantage is also due to that the different channel parameters of $H_{12}$ and $H_{21}$ when using separate arrays provide larger degrees of spatial freedom for beamforming than the case of using the same array, where the channel parameters are the same. Moreover, both ZF-Max-Power and SI-ZF-MRT are insensitive to the increase of SI, thanks to the operation of ZF SI, while the performance of SBF becomes poorer as the increase of SI, due to no operation of ZF SI. Interestingly, the JAR of LB-MMSE with separate arrays slowly decreases as the increase of SI whereas that with the same array changes little, which shows that LB-MMSE with the same array is more robust against the SI. To explain this, let us look at the third and fourth lines of Equation (25). In third line, $\frac{w_{t2}^{H}H_{21}^{H}H_{21}w_{t2}}{w_{t1}^{H}\left(I+\varepsilon_{11}H_{11}^{H}H_{11}\right)w_{t1}}$ and $\frac{w_{t1}^{H}H_{12}^{H}H_{12}w_{t1}}{w_{t2}^{H}\left(I+\varepsilon_{22}H_{22}^{H}H_{22}\right)w_{t2}}$ can be roughly seen as the receive SINRs at Node #1 and Node #2 without considering the receive AWVs, respectively. However, in order to obtain closed-form expressions of the transmission AWVs, the denominators (or numerators) of these two components are exchanged and optimized respectively, as shown in Equations (26) and (27). This means that the optimizations in Equations (26) and (27) are not to directly optimize the receive SINRs at Node #1 and Node #2. Hence, in general LB-MMSE is not so robust against the SI. However, in the case with the same array, the link from Node #1 to Node #2 is symmetric; thus the denominators (or numerators) of the two components in the third line of Equation (25) can be seen equal or at least proportional to each other. In such a case, Equations (26) and (27) are in fact to optimize the receive SINRs at Node #1 and Node #2 without considering the receive AWVs. Hence, LB-MMSE with the same array is relatively more robust against the SI.

**Figure 7:**
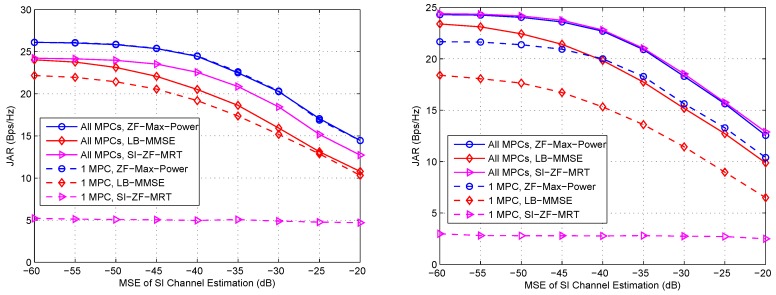
Effects of channel estimation errors on the proposed schemes with separate arrays under LOS ( **Left**) and NLOS (**Right**) channels. $\varepsilon_{12}=\varepsilon_{21}=10$ dB, $\varepsilon_{11}=\varepsilon_{22}=40$ dB, ω=π rad, d/λ=1.

Finally, we evaluate the effects of channel estimation errors on the proposed schemes. For the SI channel, there exists Gaussian error; while for the communication channel, it is possible to miss some MPCs during the beam search process. Figure 7 shows the effects of these estimation errors on the proposed schemes with separate arrays (the results are similar with the same array) under LOS and NLOS channels, respectively, where the parameters are specified in the captions. From these two figures we can observe that both ZF-Max-Power and LB-MMSE are relatively robust against the channel estimation error. Even only one MPC is acquired, they can achieve promising performance, especially under LOS channel. However, SI-ZF-MRT is not robust against the estimation error of the communication channel, i.e., if only one MPC is acquired, the performance of SI-ZF-MRT becomes rather poor, this is because the full channel information is involved in the SI ZF operation according to Equation (31).

It is noteworthy that circuit imperfections are not taken into consideration in our system model, i.e., in Equations (7) and (8). In a practical FD system, there are always Tx/Rx hardware and implementation imperfections, including low-noise amplifier (LNA) noise figure, phase noise, in-phase and quadrature (IQ) mismatch, nonlinear distortion of power amplifier, etc. These imperfections may be more severe in mmWave communication systems than in low-frequency systems, because of the higher carrier frequency and larger bandwidth. Since SI is strong, the Tx imperfections, which are carried by the transmitted signals $s_{1}$ and $s_{2}$ in Equations (7) and (8), will also arrive at the Rx. When beamforming cancellation is adopted to force the SI to zero, the performance will be affected little by the Tx circuit imperfections provided that the AWV control is perfect, because all the SI, including the imperfections, can be filtered out by beamforming. However, in practice, the AWV control may have error. In such a case the SI cannot be completely filtered out by beamforming, and the residual SI, which contains Tx imperfections, will degrade the system performance. Although baseband (BB) cancellation can be used to deal with the residual SI after beamforming cancellation, it basically cannot effectively cancel the residual Tx imperfections.

**Figure 8:**
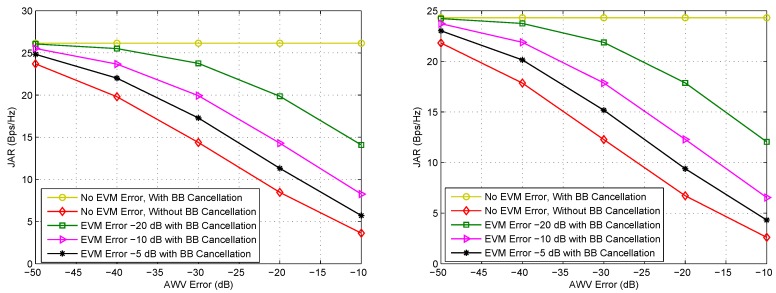
Effects of AWV error and EVM error on the JAR performance of ZF-Max-Power under LOS (**Left**) and NLOS (**Right**) channels. $\varepsilon_{12}=\varepsilon_{21}=10$ dB, $\varepsilon_{11}=\varepsilon_{22}=50$ dB, ω=0.8π rad, d/λ=1.

To further illustrate the effect of the circuit imperfections on the system performance, we model all the typical Tx imperfections as a zero-mean Gaussian distributed error vector magnitude (EVM) noise [57], and its average power can be measured in dB with respect to the transmission power. On the other hand, we also need to consider AWV control error, which can also be modeled as a zero-mean Gaussian variable, and its average power can also be measured in dB with respect to the 2-norm of its corresponding weight. By exploiting this model, we can evaluate the effects of AWV error and EVM error on the JAR performance of ZF-Max-Power as shown in Figure 8, where relevant parameters are listed in the caption. The effects are similar to the performances of the other schemes. From this figure we can find that when AWV control is perfect or AWV error is small enough, the SI as well as the EVM noise can be mitigated successfully by beamforming. However, when there is significant AWV error, the system performance deteriorates as the AWV error becomes greater. In such a case, BB cancellation is needed to cancel the residual SI. From the figure we observe that with BB cancellation, the performance is greatly improved and does not depend on the AWV error. On the other hand, BB cancellation can hardly mitigate the residual EVM noise, because it is difficult to estimate relevant parameters of the EVM noise [57]. Hence, we can observe that even when BB cancellation is adopted, if EVM noise exists, the performance still deteriorates as the AWV error becomes greater.

## 6. Conclusions

In this paper, we investigate FD mmWave communications, where we employ beamforming to cancel SI, and study a JTR-BF problem in the presence of significant SI. As the problem of finding the optimal beamforming vectors to maximize the JAR is non-convex, several suboptimal solutions are proposed. Firstly, ZF-Max-Power, which restricts the original problem by ZF SI and alternatively optimizes the desired power, is proposed, and its convergence is proven. It is shown that the computational complexity of ZF-Max-Power is lower than that of Max-SINR by one order of magnitude. Next, two closed-form solutions, namely LB-MMSE and SI-ZF-MRT are proposed, by jointly using MMSE, ZF and MRT criteria. Performance evaluations show that ZF-Max-Power approaches an upper bound on the JAR, and it needs only 2 iterations on average to achieve the convergence with random initial points. LB-MMSE and SI-ZF-MRT achieve suboptimal performances. In addition, we find that ZF-Max-Power and SI-ZF-MRT are robust against the geometry of Tx/Rx antenna arrays due to the operation of ZF SI, while LB-MMSE is not. ZF-Max-Power and LB-MMSE are robust against channel estimation error, while SI-ZF-MRT is not. Furthermore, these schemes basically achieve better JAR performance with a separate-array setting than those sharing the same array. The results demonstrate the feasibility of FD mmWave communications and the effectiveness of beamforming cancellation.

multiple

## Appendix A. Proof of Lemma 1

Since ${\left\{a_{i}\in C^{L\times 1}\right\}}_{i=1,2,\ldots ,N;\;N<L}$ is a group of linear independent vectors, they span a linear subspace denoted by $S≜S\{a_{i}{|}_{i=1,2,\ldots ,N}\}$. Suppose ${\left\{v_{i}\right\}}_{i=1,2,\ldots ,N}$ is an orthogonal basis of the linear subspace S. Then constraint $\langle a,a_{i}\rangle {=0|}_{i=1,2,\ldots ,N}$ is equivalent to $\langle a,v_{i}\rangle {=0|}_{i=1,2,\ldots ,N}$.
**Remark** *The vector* $b\in C^{L\times 1}$ *to maximize* ${\left|\langle b,a\rangle \right|}^{2}$ *with the constraint that* $\langle b,v_{i}\rangle {=0|}_{i=1,2,\ldots ,N}$ *and* ∥b∥=1 *is (before normalization)* $b=a-\sum_{i=1}^{N}\langle a,v_{i}\rangle v_{i}$.

According to the above remark, the next thing we need to prove is that $\left\{b_{i}{|}_{i=1,2,\ldots ,N}\right\}$ shown in Equation (15) is an orthogonal basis of $\left\{b_{i}{|}_{i=1,2,\ldots ,N}\right\}$. This proposition holds because $\left\{b_{i}{|}_{i=1,2,\ldots ,N}\right\}$ is obtained according to the standard Gram-Schmidt process from $\left\{a_{i}{|}_{i=1,2,\ldots ,N}\right\}$. Therefore, Lemma 1 holds.

## Appendix B. Proof of Theorem 1

Under the constraints in Equation (14), the JAR becomes

(B1) $$\begin{array}{c} R\left(w_{t1},w_{t2},w_{r1},w_{r2}\right)=\log_{2}\left(1+\varepsilon_{21}{\left|w_{r1}^{H}H_{21}w_{t2}\right|}^{2}\right)+\log_{2}\left(1+\varepsilon_{12}{\left|w_{r2}^{H}H_{12}w_{t1}\right|}^{2}\right)≜R_{\text{ub}} \end{array}$$

which shows that *R* is a function of $w_{t1},\;w_{t2},\;w_{r1}$, and $w_{r2}$.

Let $w_{t1}^{\left(n\right)}$ and $w_{t2}^{\left(n\right)}$ denote the transmit AWVs after the *n*-th iteration, and $w_{r1}^{\left(n\right)}$ and $w_{r2}^{\left(n\right)}$ the receive AWVs after the *n*-th iteration. Then we have $R^{\left(n\right)}=R\left(w_{t1}^{\left(n\right)},w_{t2}^{\left(n\right)},w_{r1}^{\left(n\right)},w_{r2}^{\left(n\right)}\right)$ and $R^{\left(n+1\right)}=R\left(w_{t1}^{\left(n+1\right)},w_{t2}^{\left(n+1\right)},w_{r1}^{\left(n+1\right)},w_{r2}^{\left(n+1\right)}\right)$.

According to Algorithms 1, as well as Equations (19) and (20), we have

(B2) $$\begin{array}{cc} & R\left(w_{t1}^{\left(n\right)},w_{t2}^{\left(n\right)},w_{r1}^{\left(n+1\right)},w_{r2}^{\left(n+1\right)}\right)\geq R\left(w_{t1}^{\left(n\right)},w_{t2}^{\left(n\right)},w_{r1}^{\left(n\right)},w_{r2}^{\left(n\right)}\right)=R^{\left(n\right)} \end{array}$$

Also, according to Algorithms 1, as well as Equations (21) and (22), we have

(B3) $$R\left(w_{t1}^{\left(n+1\right)},w_{t2}^{\left(n+1\right)},w_{r1}^{\left(n+1\right)},w_{r2}^{\left(n+1\right)}\right)=R^{\left(n+1\right)}\geq R\left(w_{t1}^{\left(n\right)},w_{t2}^{\left(n\right)},w_{r1}^{\left(n+1\right)},w_{r2}^{\left(n+1\right)}\right)$$

Therefore, we have $R^{\left(n+1\right)}\geq R^{\left(n\right)}$.

## Appendix C. Lemmas 2, 3, 4

**Lemma** **2.** *Given* $R\in C^{M\times M}$ *a positive define Hermitian matrix, the solution to maximize* $\frac{x^{H}aa^{H}x}{x^{H}\mathrm{Rx}}$ *is* $x=R^{-1}a$.

**Proof.** Since R is a positive define Hermitian matrix, $R^{1/2}$ is also an invertible Hermitian matrix. Thus, we have

(C1) $$\frac{x^{H}aa^{H}x}{x^{H}\mathrm{Rx}}=\frac{x^{H}aa^{H}x}{{\left(R^{1/2}x\right)}^{H}\left(R^{1/2}x\right)}$$

Let $\tilde{x}=R^{1/2}x$, the problem becomes to maximize $\frac{{\tilde{x}}^{H}\left(R^{-H/2}a\right){\left(R^{-H/2}a\right)}^{H}\tilde{x}}{{\tilde{x}}^{H}\tilde{x}}$. Therefore, the solution is $\tilde{x}=R^{-H/2}a=R^{-1/2}a$, i.e., $x=R^{-1}a$.  ☐

**Lemma** **3.** *Given* $R\in C^{M\times M}$ *a positive define Hermitian matrix,* $a^{H}R^{-1}a\geq \frac{{\left(a^{H}a\right)}^{2}}{a^{H}\mathrm{Ra}}$.

**Proof.** Since $R\in C^{M\times M}$ is a positive define Hermitian matrix, it can be expressed as $R=\sum_{i=1}^{M}\lambda_{i}v_{i}v_{i}^{H}$, where $\lambda_{i}>0$ and ${\left\{v_{i}\right\}}_{i=1,2,\ldots ,M}$ constitutes an orthogonal base in $C^{M\times M}$. Thus, a can be expressed as $a=\sum_{i=1}^{M}\alpha_{i}v_{i}$. Besides, we have $R^{-1}=\sum_{i=1}^{M}\frac{1}{\lambda_{i}}v_{i}v_{i}^{H}$. Thus,

(C2) $$\begin{array}{cc} & \left(a^{H}R^{-1}a\right)\left(a^{H}\mathrm{Ra}\right)\\ = & \left(\sum_{i=1}^{M}\frac{{\left|\alpha_{i}\right|}^{2}}{\lambda_{i}}\right)\left(\sum_{i=1}^{M}{\left|\alpha_{i}\right|}^{2}\lambda_{i}\right)\\ = & \sum_{i=1}^{M}{\left|\alpha_{i}\right|}^{4}+\sum_{i=1}^{M}\sum_{j=1}^{i-1}{\left|\alpha_{i}\right|}^{2}{\left|\alpha_{j}\right|}^{2}\left(\frac{\lambda_{j}}{\lambda_{i}}+\frac{\lambda_{i}}{\lambda_{j}}\right)\\ \geq & \sum_{i=1}^{M}{\left|\alpha_{i}\right|}^{4}+2\sum_{i=1}^{M}\sum_{j=1}^{i-1}{\left|\alpha_{i}\right|}^{2}{\left|\alpha_{j}\right|}^{2}\\ = & {\left(a^{H}a\right)}^{2} \end{array}$$

☐

**Lemma** **4.** *Given* $R\in C^{M\times M}$ *a positive define Hermitian matrix with the formulation* $R=I+\varepsilon bb^{H}$, $\frac{a^{H}a}{a^{H}\mathrm{Ra}}\geq \frac{1}{1+\varepsilon b^{H}b}$.

**Proof.** Let v=a/∥a∥. We have

(C3) $$\begin{array}{cc} & \frac{a^{H}a}{a^{H}\mathrm{Ra}}=\frac{a^{H}a}{a^{H}\left(I+\varepsilon bb^{H}\right)a}=\frac{1}{v^{H}\left(I+\varepsilon bb^{H}\right)v}\\ = & \frac{1}{1+\varepsilon {\left|b^{H}v\right|}^{2}}\geq \frac{1}{1+\varepsilon {\left|\frac{b^{H}b}{∥b∥}\right|}^{2}}=\frac{1}{1+\varepsilon b^{H}b} \end{array}$$

☐
